# New Vistas in *Mycobacterium tuberculosis* Infection and Its Association with Lung Cancer Development

**DOI:** 10.3390/cancers17132224

**Published:** 2025-07-02

**Authors:** Kostas A. Papavassiliou, Amalia A. Sofianidi, Fotios G. Spiliopoulos, Alice G. Vassiliou, Athanasios G. Papavassiliou

**Affiliations:** 1 First University Department of Respiratory Medicine, ‘Sotiria’ Chest Hospital, Medical School, National and Kapodistrian University of Athens, 11527 Athens, Greece; konpapav@med.uoa.gr; 2 Department of Biological Chemistry, Medical School, National and Kapodistrian University of Athens, 11527 Athens, Greece; amaliasof@med.uoa.gr (A.A.S.); spiliopoulosfotis99@gmail.com (F.G.S.); 3 First Department of Critical Care Medicine, ‘Evangelismos’ Hospital, Medical School, National and Kapodistrian University of Athens, 10676 Athens, Greece; alvass75@gmail.com

Lung cancer is the leading cause of cancer-related mortality worldwide, with the American Cancer Society estimating approximately 124,730 deaths from lung cancer in 2025 [1]. Another major public health issue is tuberculosis (TB), an infection caused by *Mycobacterium tuberculosis* (MTB). According to the World Health Organization (WHO), lung cancer ranked as the sixth leading cause of death in 2021, while tuberculosis ranked as the tenth [2]. The oncogenic potential of various infectious agents has been established for years; multiple viruses and microbes, such as the bacterium *Helicobacter pylori*, are listed amongst them [3]. A volume of data suggest that there is a causal relationship between previous MTB infection and lung cancer development [4]. In addition, the oncogenic nature of non-tuberculous *Mycobacterium* complex infections has already been described; *Mycobacterium leprae*, *Mycobacterium ulcerans*, and *Mycobacterium xenopi* are incriminated in augmented risk of tumorigenesis in different organs [5]. However, the molecular and pathophysiological mechanisms underlying the connection between MTB and lung cancer remain largely unknown. Herein, we provide a succinct overview of recent research shedding light on TB infection and subsequent lung carcinogenesis.

Although the International Agency for Research on Cancer (IARC) has not yet classified MTB as a carcinogenic agent [6], there are several epidemiological reports indicating heightened lung cancer risk in patients with previous TB infection [7,8]. A recently published meta-analysis confirmed a statistically significant link between a previous pulmonary TB infection and lung neoplasm formation (odds ratio (OR) 2.09, 95% confidence interval (CI) 1.62–2.69, *p* < 0.001) [4]. Additionally, a population-based study in Taiwan demonstrated that the risk of pulmonary malignancy development was 1.67-fold higher in the group of patients with a medical history of pulmonary TB disease than in the group of patients with an unremarkable medical history [9].

The precise molecular underpinnings of lung tumorigenesis due to MTB infection are yet to be elucidated. It is clear that reactive oxygen species (ROS) alongside reactive nitrogen species (RNS) are essential for the initiation and the sustenance of carcinogenesis [10]. Macrophages and neutrophils, cells aberrantly involved in MTB infection, produce the aforementioned harmful species leading to perturbation of redox homeostasis, which represents a major hallmark of cancer development. An inflammatory environment in the lung is created, which results in persistent tissue impairment due to the prolonged nature of MTB infection [11,12]. Furthermore, long-term inflammation is associated with DNA damage, which eventually triggers lung tumorigenesis [13]. Also, tenacious inflammation gives rise to fibrosis and scar formation, elements with high tumorigenic potential [14].

Delving deeper into the intersection between MTB infection and lung cancer, it has recently been proposed that bacterial DNA could be incorporated into the DNA of bronchial epithelial cells, causing their malignant transformation [15]. This mechanism is not new; hepatitis B virus has been implicated in liver carcinogenesis by integrating into the hepatic genome [16]. Indeed, the IS6110 MTB transposon, widely recognized for its utility in TB diagnosis and epidemiology, was detected inside the nucleus of cancer cells derived from non-small-cell lung cancer (NSCLC) patients. The IS6110 transposon is unique for MTB and its presence in the host genome verifies bacterial DNA integration [17]. Moreover, granuloma formation, which is the “stamp” of MTB infection, is fulfilled through the induction of epithelial cadherin (E-cadherin) expression by macrophages. E-cadherin expression, a Ca^2+^-dependent cell–cell adhesion transmembrane glycoprotein, confers an epithelioid phenotype to macrophages via a process termed mesenchymal–epithelial transition (MET) [18]. On the other hand, MTB prompts epithelial–mesenchymal transition (EMT), a major hallmark of cancer verified in lung cancer cell lines [19]. Notably, both MET and EMT are processes involved in tumor initiation alongside tumor metastasis [20].

Secondary lung cancer due to MTB infection involves the regulation of genes that foster tumorigenesis. MTB induces DNA damage through the SecA2 secretome, which leads to activation of the DNA damage response (DDR) pathway, and particularly the stimulation of the host ataxia telangiectasia mutated (ATM) serine/threonine kinase. The ensuing cell cycle alteration inhibits apoptosis and accentuates cell growth [21]. A recent study demonstrated that a vital enzyme engaged in MTB infection development, namely protein phosphatase 2A (PP2A) phosphatase activator (PtpA), influences the expression of the antigen Kiel 67 (Ki-67; also known as marker of proliferation Kiel 67 (MKI67)) gene. MKI67 encodes the Ki-67 protein, a prominent tumor cell marker correlated with various stages of tumorigenesis [22]. Another study utilizing bioinformatics revealed a different expression pattern of the Keratin 80 (KRT80; a typical epithelial cell marker) gene in TB versus lung adenocarcinoma. KRT80 exhibited opposite expression patterns between the two diseases; it was downregulated in TB but upregulated in NSCLC. This differential expression suggests a potential shift in the role of the KRT80 gene during disease progression from TB infection to lung cancer, which likely promotes oncogenesis [23]. Likewise, differences in the frequency of epidermal growth factor receptor (EGFR) mutations between patients with TB and lung cancer have been studied. It has been shown that patients with previous TB infection have a higher rate of EGFR mutations and worse treatment responses to EGFR tyrosine kinase inhibitors (TKIs) in case they develop lung cancer [24]. Apparently, further research is needed in the field, as these observations could potentially change the therapeutic algorithms that are currently used in clinical lung cancer practice.

The updated hallmarks of cancer, introduced by Hanahan in 2022, recognized the modulatory role of the microbiome in tumorigenesis [25]. DNA damage and host immune response modification are listed amongst the mechanisms employed by the microbiome to stimulate malignant transformation of cells [25]. In this vein, it has been proposed that in the case of TB infection, prolonged antibiotic administration disrupts microbial living in the gut, triggering a microbiome-impelled immunomodulation that drives lung cancer growth [26]. Whether probiotics could help soothe these responses and eliminate the risk of lung cancer evolution is an area of future exploration suggested by investigators [26]. As far as immune destruction avoidance by malignant cells is concerned, it has been a well-established cancer hallmark since 2011 [27]. Immune suppression has also emerged as a hallmark of MTB infection; MTB exploits the programmed cell death protein 1 (PD-1)/programmed death-ligand 1 (PD-L1) pathway to promote lung cancer metastasis and conquer the cellular immune response supported by Th1 cells. Immune suppression renders cancer development and progression unchallenging [28]. Although this observation was made in preclinical models, it can be concluded that the PD-1/PD-L1 pathway could be therapeutically exploited earlier on during TB lung infection to prevent future oncogenesis.

In summary, TB lung infection and lung cancer are two entities that have intricate connections with one another. TB has proven to be a major risk factor for lung carcinogenesis; lung inflammation induced by MTB leads to tissue lesion and subsequent scar formation and DNA damage, processes that encourage neoplasia. Extensive research is urgent to unravel the complexity underlying this perplexing relationship and move towards prevention rather than cure. Evidently, medical practitioners should be aware of distinguishing these two diseases and be alert to patients with a history of TB infection in case they develop early clinical signs of lung cancer.
